# Cynomolgus monkeys (*Macaca fascicularis*) experimentally and naturally infected with hepatitis E virus: The bone marrow as a possible new viral target

**DOI:** 10.1371/journal.pone.0205039

**Published:** 2018-10-02

**Authors:** Fernanda de Oliveira Bottino, Noemi Rovaris Gardinali, Sarah Beatriz Salamene Salvador, Andreza Soriano Figueiredo, Lynn Barwick Cysne, Juliane Siqueira Francisco, Jaqueline Mendes de Oliveira, Marcelo Pelajo Machado, Marcelo Alves Pinto

**Affiliations:** 1 Laboratory of Technological Development in Virology, Oswaldo Cruz Institute, Oswaldo Cruz Foundation, Rio de Janeiro, Brazil; 2 Institute of Science and Technology of Biomodels, Oswaldo Cruz Foundation, Rio de Janeiro, Brazil; 3 Laboratory of Pathology, Oswaldo Cruz Institute, Oswaldo Cruz Foundation, Rio de Janeiro, Brazil; Centre de Recherche en Cancerologie de Lyon, FRANCE

## Abstract

Hepatitis E virus (HEV) transmission through infected blood and blood products has already been described. However, little is known about the bone marrow (BM) as source of HEV infection. Our study aimed to investigate the presence of HEV antigen (Ag) and histological changes in BM of cynomolgus monkeys (*Macaca fascicularis*) experimentally and naturally infected with HEV. Four cynomolgus monkeys with acute, and two with chronic hepatitis E ─ after immunosuppressive therapy with tacrolimus ─ were compared with one colony-bred animal naturally infected. Both, natural and experimental infections were characterized by anti-HEV IgG seroconversion detected by ELISA, and viral RNA isolation confirmed by RT-qPCR and qualitative nested RT-PCR. BM biopsies were collected from all animals, submitted to histology and indirect immunofluorescence techniques and observed, respectively, by light and confocal microscopy. The HEV Ag-fluorescent-labeled cells were detected from BM biopsies obtained from three monkeys with acute and one with chronic hepatitis E, and also from the naturally infected monkey. In the experimentally infected animals with acute hepatitis, HEV Ag detection occurred at 160 days post-infection, even after viral clearance in serum, feces, and liver. Double-stranded RNA, a replicative marker, was detected in BM cells from both acute and chronically infected animals. Major histological findings included vacuolization in mononuclear and endosteal cells, an absence of organized inflammatory infiltrates, and also some fields suggesting displasic focal BM disease. These findings support the hypothesis of BM cells as secondary target sites of HEV persistence. Further experimental studies should be carried out to confirm the assumption of HEV transmission through BM transplantation.

## Introduction

Hepatitis E virus (HEV) infection is a major cause of acute viral hepatitis worldwide [1]. Every year, there are an estimated 20 million new human cases, more than three million symptomatic cases and 44,000 disease-related deaths [1]. HEV is classified in family *Hepeviridae*, genera *Orthohepevirus A*, lately grouped in five genotypes infecting humans (1, 2, 3, 4 and 7) and three genotypes infecting wild boars (5 and 6) and Bactrian camels (8) [2–5]. HEV genotypes 1 and 2 are endemic in developing countries where transmission occurs mainly via the fecal-oral route and contaminated water is the main source of infection [6]. Genotypes 3 (HEV-3) and 4 (HEV-4) are prevalent in developed countries where autochthonous cases of acute HEV infection are attributed mainly to zoonotic transmission to humans, associated with consumption of raw or undercooked meat from pigs, wild boars and other mammals [7, 8].

Further, human cases of persistent HEV-3 infection evolving to chronic hepatitis were described in solid organ transplant (SOT) patients under immunosuppressive therapy with, e.g. tacrolimus, considered a potent macrolide immunosuppressant derived from *Streptomyces tsukubaensis* (calcineurin pathway inhibitor) and a first-line medication employed to reduce the rate of rejection, especially in parenchymal organ transplantation [9]. High doses of tacrolimus showed to promote infection of liver cells with HEV in cell culture models [10]. It is considered a risk factor for virus persistence in the host [11, 12]. Also, occurrence of HEV-3 related chronic hepatitis was reported in recipients of allogeneic bone marrow transplantation [13–15]. Besides, Hepatitis-Associated Aplastic Anemia (HAAA) and Diamond-Blackfan Anemia (DBA), as well as some unspecific hematological changes, such as lymphopenia and leukopenia, were reported in patients with hepatitis E [16–19].

Recent findings, such as detection of HEV in allogeneic hematopoietic stem cell transplantation (alloHSCT) patients, gave rise to concerns [15]. Also, hematopoietic stem cells (HSC) donors were shown to harbor HEV infections [20–22]. Therefore, many authors are suggesting HEV screening as routine in blood banks and transplantation registries [15, 20–24]. However, whether BM allogeneic transplants can be a potential source of HEV transmission to recipients is yet unclear.

Cynomolgus (*Macaca fascicularis*) is the best model to mirror human organ transplantation [25, 26]. Besides, it is considered the primary model for studying acute and chronic clinical course of HEV infection, as confirmed by our group [12].

In this context, the aims of our study were (i) to investigate the presence of HEV Ag in bone marrow cells from two groups of cynomolgus monkeys infected experimentally, with acute and chronic hepatitis E; (ii) to determine the anti-HEV seroconversion profile using different point-in-time samples from a colony-bred cynomolgus monkey infected naturally with HEV and (iii) to compare detection of HEV Ag and histological findings of these three groups of HEV-infected animals.

## Materials and methods

### Ethics statement

All monkeys obtained for this study originated from a breeding colony from the Institute of Science and Technology in Biomodels (ICTB), of the Oswaldo Cruz Foundation (Fiocruz), Rio de Janeiro, Brazil. Animals submitted to the experimental infection were kept in Biohazard Level 2 facilities in a single house in stainless steel squeeze-back cages (0.77 m height x 0.60 m width x 0.68 m depth) in a climate-controlled room (temperature of 22 ± 1°C and humidity 55 ± 5%) with a 12h light / dark cycle. Those animals were euthanized by exsanguination (cardiac puncture) under deep barbiturate anesthesia with sodium thiopental 2.5% at 25 mg/kg (Thiopentax, Cristalia, São Paulo, Brazil), which was delivered intravenously. The colony-bred animal naturally HEV infected was housed outdoor in a big cage (6 m x 6.4 m x 4.2 m), together with other ten cynomolgus monkeys.

All animals were fed daily with a commercial primate diet supplemented with vegetables and fresh fruits. Water was provided *ad libitum*. Environmental enrichment programs, such as: alimentary (popcorn and nuts), audio-visual (audios with forest themes and movies), and tactile enrichment (toys such as hanging balls) were offered throughout the study.

Clinical procedures and samples collection were performed under anesthesia with ketamine hydrochloride at 20 mg/kg (Vetanarcol, Konig, Argentina) combined with midazolam at 0.1 mg/kg (Cristalia, Rio de Janeiro, Brazil). To reduce post-operatory pain, 0.2 ml of 2% lidocaine was injected subcutaneously at bone marrow (BM) puncture site.

The housing standard adopted in our study attended to space recommendations for individually NHP with a maximum weight of 7 kg, in accordance with the Brazilian Normative Resolution CONCEA n.28, of November 13, 2015 (http://www.mct.gov.br/upd_blob/0240/240230.pdf). Experimental protocols were approved (LW5/16 and LW-17/13) by the Institutional Animal Care and Use Committee (CEUA-Fiocruz), and conducted in strict accordance with the recommendations from the Guide for Care and Use of Laboratory Animals of the Brazilian Society of Science in Laboratory Animals (SBCAL) and the National Council for the Control of Animal Experimentation (CONCEA, Brazil).

### Animals and study design

In order to evaluate the hypothesis of BM cells as reservoirs of HEV, biopsies were performed in iliac crest from six cynomolgus monkeys HEV infected experimentally: four with acute and two with chronic hepatitis E, as previously described by our group (Table 1) [12]. HEV acute hepatitis was defined by absence of inflammatory infiltrates before 160 days post-infection (dpi), and sustained virological response before 69 dpi. At the end of the experiment (160 dpi), chronic hepatitis was characterized by histology as a limited area of interface hepatitis (piecemeal necrosis), associated with the detection of HEV RNA in serum, feces and liver. All animals that developed chronic hepatitis E were previously treated with tacrolimus. Histological analysis and immunostaining for detection of HEV Ag were performed in BM biopsies.

**Table 1:** Gender, age, sex, body weight, and hepatitis E course of cynomolgus monkeys experimentally infected with HEV.

Monkey ID*	Gender	Age (year)	Weight (kg)*	Hepatitis E course
**V12**	Female	8	3.10	Acute
**AC11**	Male	3	3.07	Acute
**AE3**	Male	1	1.55	Acute
**AD8**	Female	2	1.83	Acute
**AB19**	Male	5	3.50	Chronic
**AE6**	Female	1	1.30	Chronic

*ID, identification; kg, kilogram

Table adapted from Gardinali *et al*. (2017) [12]

**Fig 1:**
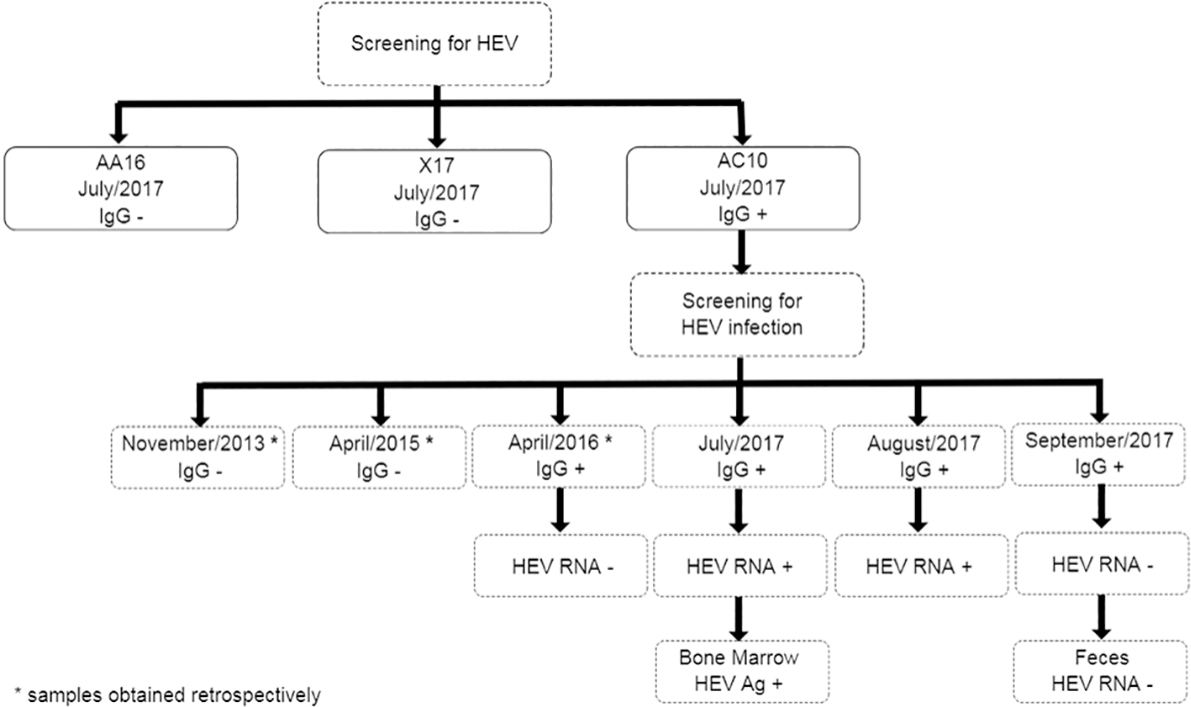
Overview of cynomolgus monkey’s screening with samples collection date, and study results.

Aiming to compare natural and experimental infections, histological and HEV Ag profiles were also evaluated using BM samples from an adult cynomolgus monkey infected naturally. This animal was noted as strongly reactive for anti-HEV IgG under routine screening of three cynomolgus monkeys from a NHP research facility of the ICTB / Fiocruz (Table 2). Aiming to confirm natural HEV infection, three earlier and two subsequent serum samples were tested for detection of anti-HEV IgG and HEV RNA (Fig 1).

**Table 2:** Gender, age, sex, body weight, and anti-HEV IgG detection of colony-bred cynomolgus monkeys.

Monkey ID*	Gender	Age (year)	Weight (kg)*	Anti-HEV IgG*
**AA16**	Female	9	2.70	**-**
**X17**	Male	10	5.74	**-**
**AC10**	Female	6	3.09	**+**

*ID, identification; kg, kilogram; IgG, immunoglobulin

### Bone marrow biopsy and blood collection

BM biopsies were obtained aseptically from the iliac crest using a 14G needle (Ítaca Laboratórios Ltda, Brazil). The interpretable biopsy lengths were 3 to 5 mm with a diameter of 2.1 mm. When the fragments were too small, two to three perforations were performed. Samples were obtained at 80 days pre-infection (T0) and 160 dpi (T1) from both groups of animals, with acute and chronic hepatitis E. With the naturally infected cynomolgus monkey (AC10) similar procedures were adopted for BM biopsy. Blood samples were collected from femoral vein. Serum samples were stored at -20°C for serological tests and -70°C for virological analysis.

### Histological analysis

BM biopsies were fixed in 10% formalin, maintained in 10% EDTA decalcifying solution (0.1 M phosphate buffer) and processed according to standard histological techniques for paraffin embedding, as follow: 70% Ethanol, one change, 1 hour; 80% Ethanol, one change, 1 hour; 95% Ethanol, one change, 1 hour; 100% Ethanol, three changes, 1.5 hour each; Xylene, two changes, 1.5 hour each; Paraffin wax (58‐60°C), two changes, 2 hours each; Embedding tissues into paraffin blocks.

Tissue sections (5 μm) were stained with hematoxylin and eosin [27] and analyzed in an Axiovert Z1 brightfield microscope (Carl Zeiss, Germany) equipped with a mRC5 Axiocam digital camera (Carl Zeiss, Germany).

### HEV antigen detection

Paraffin-shaped BM sections (5μm) were examined by indirect immunofluorescence using a mouse monoclonal antibody that recognizes HEV ORF2 antigen (IgG2a, 1mg/ml) [clone ab101124] (Abcam, UK) at 1:50 dilution and a mouse monoclonal antibody that recognizes double-strand RNA (IgG2a, 1mg/ml) [clone J2] (Scicons, Hungary) at 1:100 dilution. A goat anti-mouse polyclonal antibody conjugated with Alexa Fluor 488 (IgG, 2mg/ml) [cat: A32723] (Thermofisher, USA) was used as a secondary antibody. Antigenic retrieval was carried out in 0.01 M citrate buffer pH 6.0 in Pascal chamber (Dako, USA), according to the manufacturer’s instructions. Thereafter, a counter-staining with DAPI 1:5000 [cat: 03571] (Molecular Probes, USA) and a background staining with Evans Blue 1:10000 were performed. Negative controls were performed by duplicating each sample and omitting treatment with the primary antibodies, so that any reactions resulting from the secondary antibodies or reagents employed in the analyses could be adequately traced. Slides were mounted with ProlongGold Antifade [cat: P36934] (Life Technologies, USA) and analyzed using LSM 710 Confocal Laser Scanning Microscope (Carl Zeiss, Germany).

### Serological assays

Serological detection of anti-HEV IgG antibodies was performed using the commercially available kit recomWell HEV IgG (Mikrogen Diagnostik, Germany) according to the manufacturer’s instructions.

### Biochemical analysis

Serum alanine aminotransferase (ALT) and aspartate aminotransferase (AST) levels were determined by *Vitros* DT60 II chemistry system (Johnson & Johnson's, Minnesota, USA).

### RNA extraction, nested RT-PCR and RT-qPCR

HEV RNA was extracted from serum samples and 10% w/v fecal suspensions using QIAamp viral RNA mini kit (QIAGEN, Hilden, Germany) according to the manufacturer’s instructions. Reverse transcription (RT) and PCR reactions were performed in a single step using the SuperScript III One-Step RT-PCR System with Platinum Taq DNA Polymerase (Invitrogen Life Technology, USA). RT-PCR and nested RT-PCR were performed using a set of primers targeting ORF2 region, as previously described [28]. RT-qPCR was performed using AgPath-ID one-step RT-PCR kit (Applied Biosystems, USA) using primers and probe previously described [29].

### Sequencing reactions and phylogenetic analysis

The amplification product of ORF2 was purified using reagents and protocols of the commercial Wizard SV Gel kit and PCR Cleaning System (Promega, USA). Sequencing reactions were performed using reagents and protocols of Big Dye Terminator 3.1 kit (Applied Biosystems, EUA). Phylogenetic analyses were conducted with Bayesian inference using Markov Chain Monte Carlo (MCMC) statistical framework implemented in the program BEAST v1.8.1 [30] under TRN+G nucleotide substitution model. A phylogenetic tree, based on the HEV ORF2 region (302bp), was constructed with sequences retrieved from GenBank, including prototype sequences from HEV genotypes 3 and 4.

## Results

**Fig 2:**
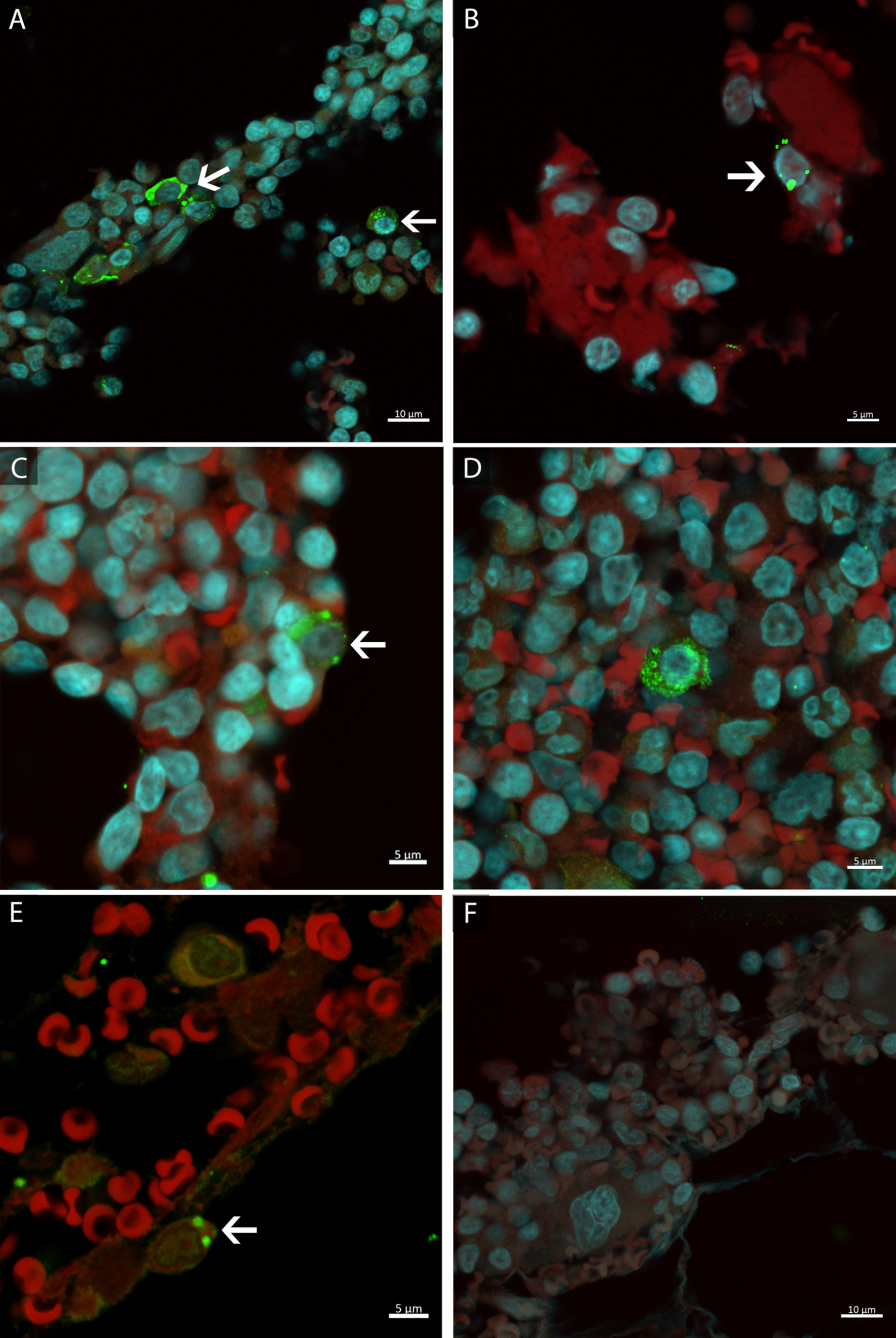
Bone marrow cells of cynomolgus monkeys with hepatitis E virus infection at 160 dpi. HEV antigen detection in: **(A)** one monkey with chronic hepatitis E; **(B-D)** three monkeys with acute hepatitis E; **(E)** one monkey naturally HEV infected. **(F)** Negative results in a monkey chronically infected. HEV antigen detection (→) in green, nuclei stained with DAPI in blue and erythroid cells and background in red.

HEV Ag-labelled bone marrow (BM) cells were detected in three out of four cynomolgus with acute hepatitis E (AE3, AC11, and AD8) and in one out of two with chronic hepatitis E (AE6) at 160 dpi (Fig 2A–2D;2F) (Table 3). These cells presented a dotted-shape green labelling (HEV Ag-positive), sometimes spread through the cytoplasm (Fig 2A;2D), sometimes concentrated in a few inclusions (Fig 2B;2C). BM samples collected before HEV infection (T0) did not show specific labeled-cells (Table 3).

**Table 3:** HEV antigen detection in bone marrow biopsies from animals infected experimentally.

Monkeys ID*	Hepatitis E Course	HEV Ag (T0)*	HEV Ag (T1)*	dsRNA*
**V12**	Acute	**-**	**-**	NA*
**AC11**	Acute	**-**	**+**	NA*
**AE3**	Acute	**-**	**+**	+
**AD8**	Acute	**-**	**+**	+
**AB19**	Chronic	**-**	**-**	NA*
**AE6**	Chronic	**-**	**+**	+

*ID, identification; HEV Ag, hepatitis E virus antigen; T0,80 days pre-infection; T1, 160 dpi; dsRNA, double-stranded RNA; NA, not available

**Fig 3:**
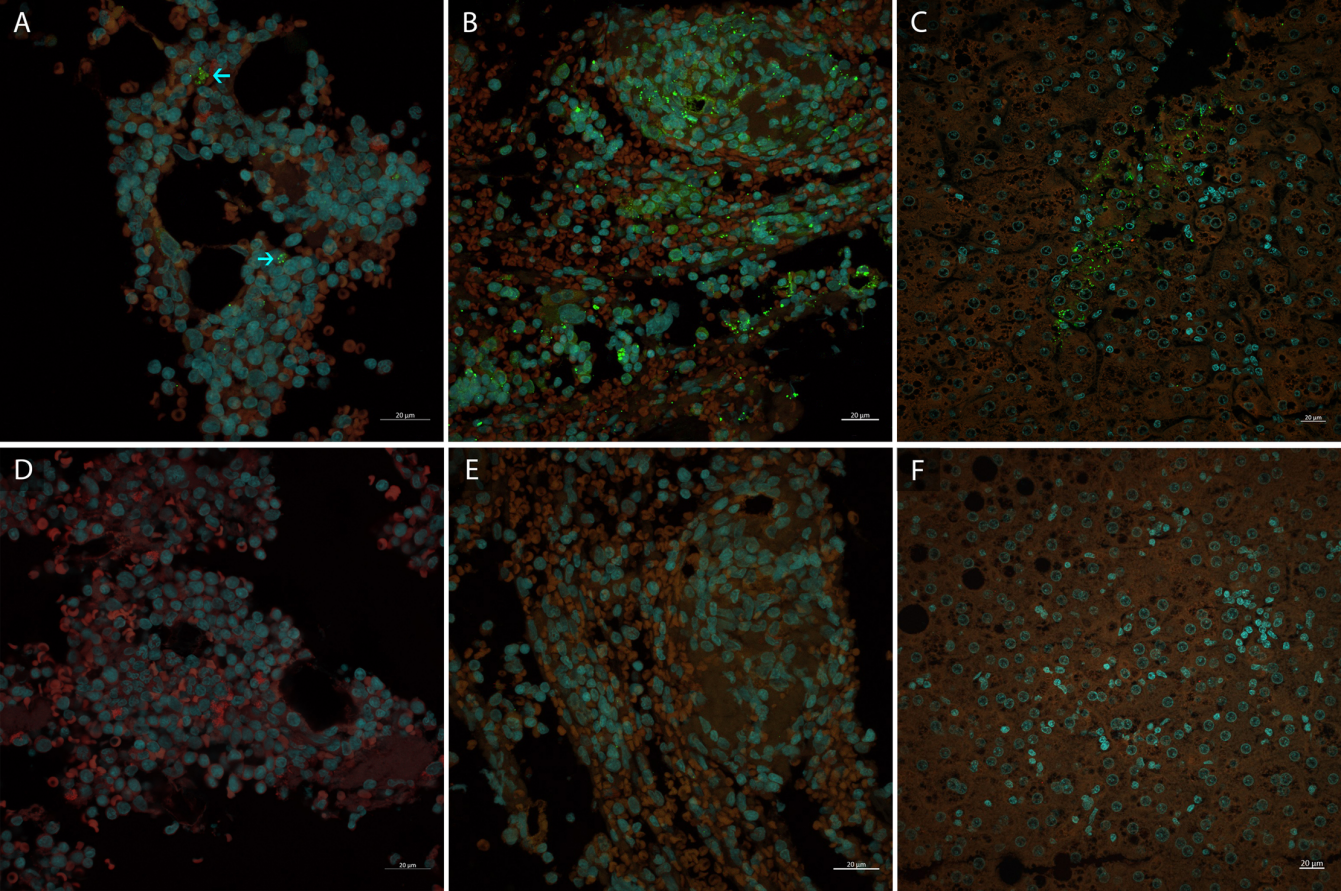
Immunofluorescence analysis of dsRNA detection from cynomolgus monkeys with hepatitis E virus infection at 160 dpi. Bone marrow cells of **(A)** one monkey with acute hepatitis E and **(B)** one monkey with chronic hepatitis E; **(C)** liver of one monkey with chronic hepatitis E. **(D-F)** Negative controls omitting the primary antibody. HEV antigen detection (→) in green, nuclei stained with DAPI in blue and erythroid cells and background in red.

Double-stranded RNA (dsRNA) was detected at 160 dpi, from both acute and chronically infected animals, by immunostaining (Fig 3A;3B) (Table 3). The pattern of dsRNA labeled-cells was similar to those observed in the liver of the chronically infected monkey witch was found to be positive for negative strand HEV RNA by RT-PCR (Fig 3C). The frequency of labeled cells was highlighted in chronically HE-infected monkeys. Negative controls (omitting the J2 primary antibody) did not show specific labeled-cells (Fig 3D–3F)

**Fig 4:**
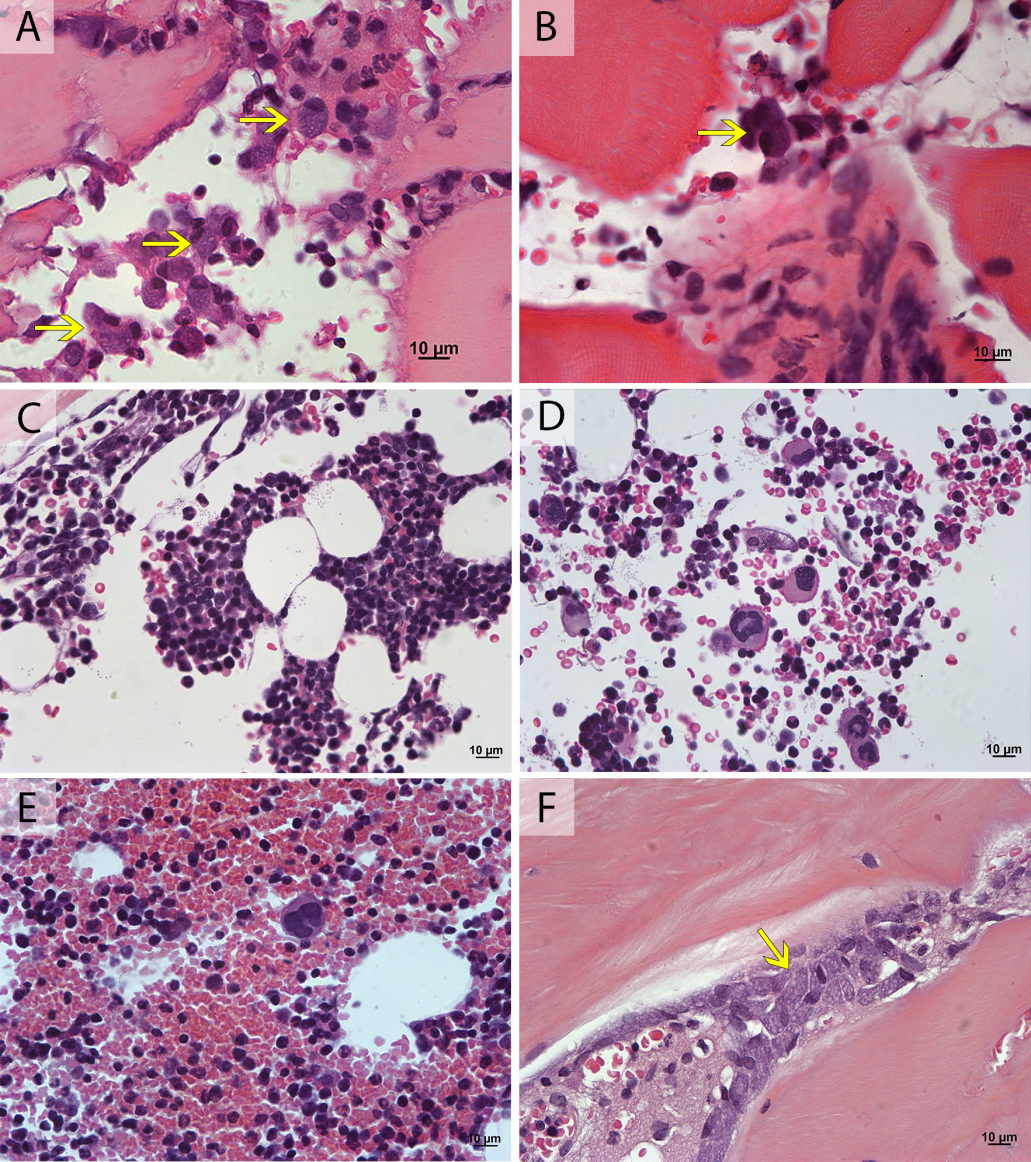
Brightfield microscopy of bone marrow biopsies of cynomolgus monkeys experimentally infected with hepatitis E virus at 160 dpi. Histological analysis showing: **(A-B)** vacuolization in mononuclear cells (→); **(C)** lymphocyte proliferation and activation clusters; **(D)** megakaryocytosis (>5 megakaryocytes/field); **(E)** absence of megakaryocytosis (0–2 megakaryocytes/field); and **(F)** vacuolization in endosteal cells (→). Hematoxilin and Eosin stain.

Histological analysis of BM, at 160 dpi, from all HEV Ag-positive monkeys (acute and chronic hepatitis E) revealed vacuolated mononuclear cells (Fig 4A;4B). Also, mononuclear cells spread within BM parenchyma did not show organized inflammatory infiltrates. Clusters of lymphocyte proliferation and activation (Fig 4C), as well as megakaryocytosis (>5 megakaryocytes / field) (Fig 4D) were observed in a single animal with acute hepatitis E (AE3). The other animals showed megakaryocytes counts similar to those observed in pre-inoculation step (0–2 megakaryocytes / field) (Fig 4E). Vacuolization in endosteal cells was observed in both groups, acute and chronic (Fig 4F) (Table 4).

**Table 4:** Summary of bone marrow histological changes and HEV antigen detection in naturally and experimentally HEV infected cynomolgus monkeys.

	Megakaryocytosis	Lymphocyte activation and proliferation clusters		Vacuolated cells	HEV Ag* detection
*Chronically experimentally HEV infected monkeys*					
**AB19**	**-**	**-**		**-**	**-**
**AE6**	**-**	**-**		**+**	**+**
*Acutely experimentally HEV infected monkeys*					
**V12**	**-**	**-**		**-**	**-**
**AC11**	NA*	**-**		**+**	**+**
**AE3**	**+**	**+**		**+**	**+**
**AD8**	**-**	**-**		**+**	**+**
*Naturally HEV infected monkey*					
**AC10**	**-**	**-**		**-**	**+**

*HEV Ag, hepatitis E virus antigen; NA, not analyzed (small area available for analysis)

**Fig 5:**
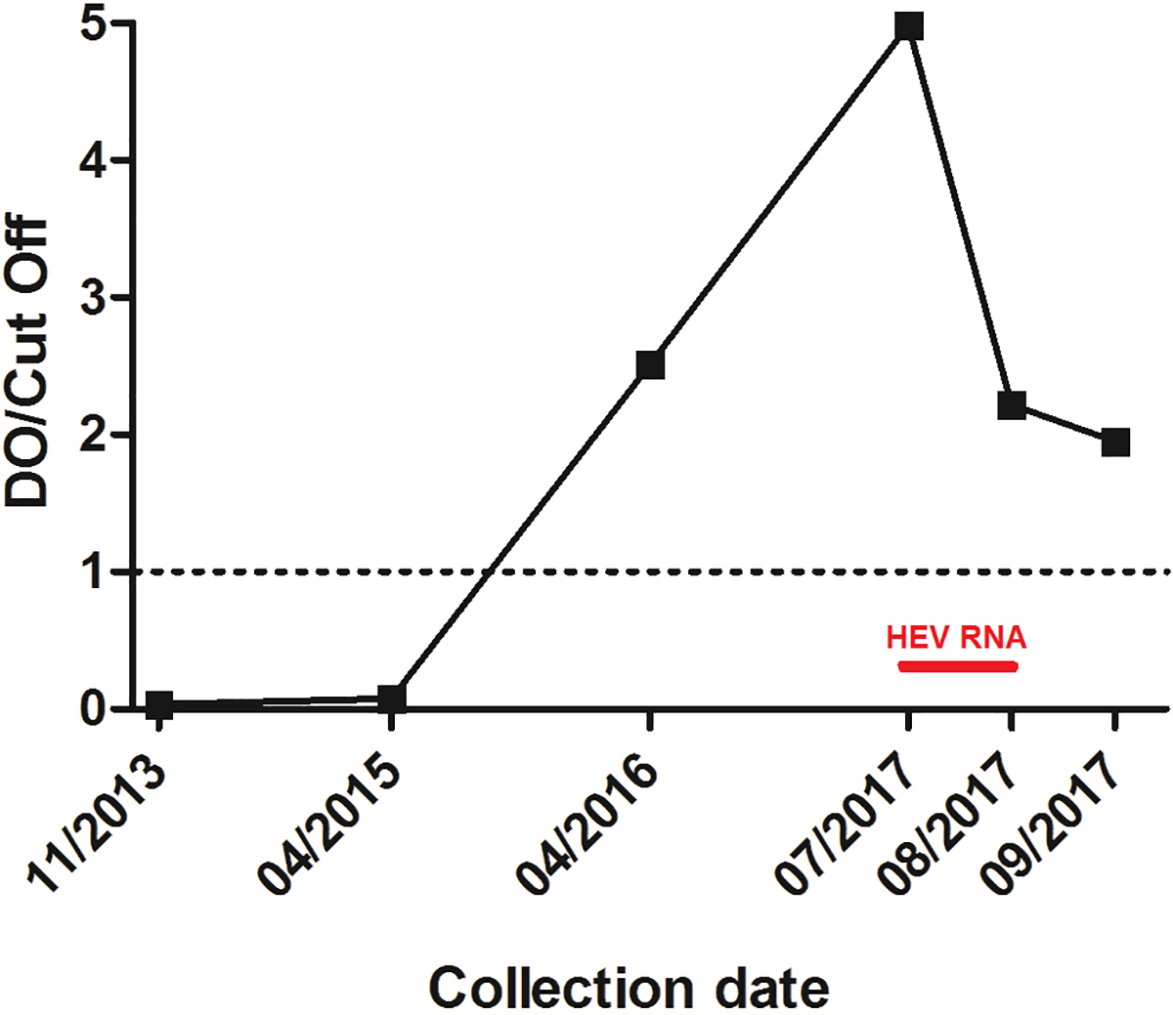
Anti-HEV IgG seroconversion and HEV RNA detection from the monkey naturally infected, between 2013 and 2017. Samples with OD/cutoff ratios above 1.0 are considered positive for anti-HEV IgG.

**Fig 6:**
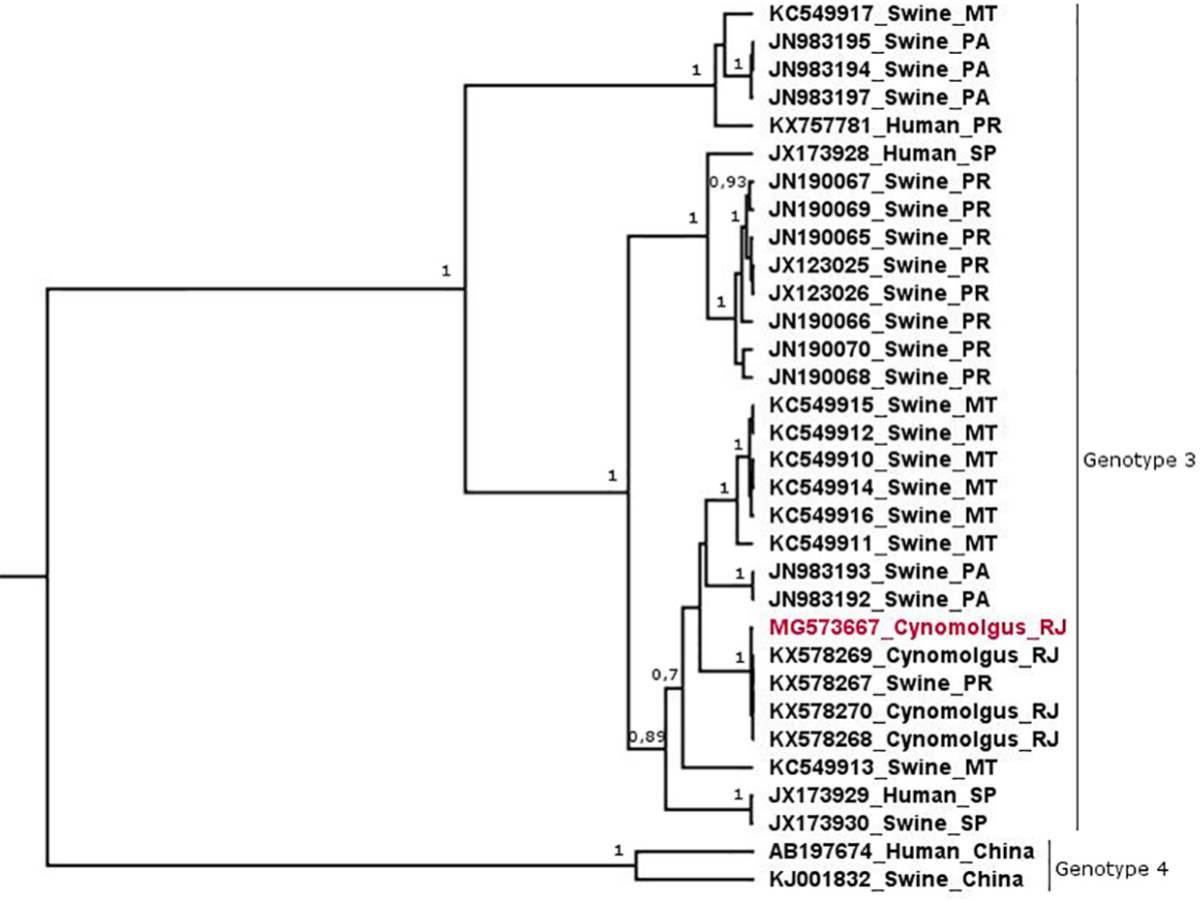
Phylogenetic analysis of HEV strains using a partial nucleotide sequence of ORF2 (302bp). Phylogenetic tree was constructed by Bayesian inference using the Bayesian Markov chain Monte Carlo (MCMC) statistical framework implemented in the BEAST v1.8.1 program [30], under the TRN + G replacement model. The posterior probability values ​​(pp) are at the beginning of each clade. The sequences used are indicated with the GenBank accession number, host and Brazilian state of origin. The sequence obtained in this study is highlighted in red.

The naturally infected monkey (AC10) showed typical serological and virological changes, as previously described by our group [31]. Results are summarized in Fig 1. Anti-HEV IgG seroconversion was confirmed by ELISA, and HEV RNA was detected by RT-PCR and RT-qPCR in serum samples (Fig 5). Serum levels ​​of ALT and AST were within the normal range for the species, 27 and 59 IU/L, respectively. Phylogenetic reconstruction using a partial nucleotide (nt) sequence (ORF2, 302 nt) revealed that AC10 HEV isolate belongs to HEV genotype 3 (Fig 6). The nucleotide sequence shared 99% identity with chronically infected cynomolgus isolates from our experimental infection study (accession numbers: KX578268, KX578269, and KX578270) [12]. The partial genomic sequence reported in this study was deposited in the GenBank under the accession number MG573667.

**Fig 7:**
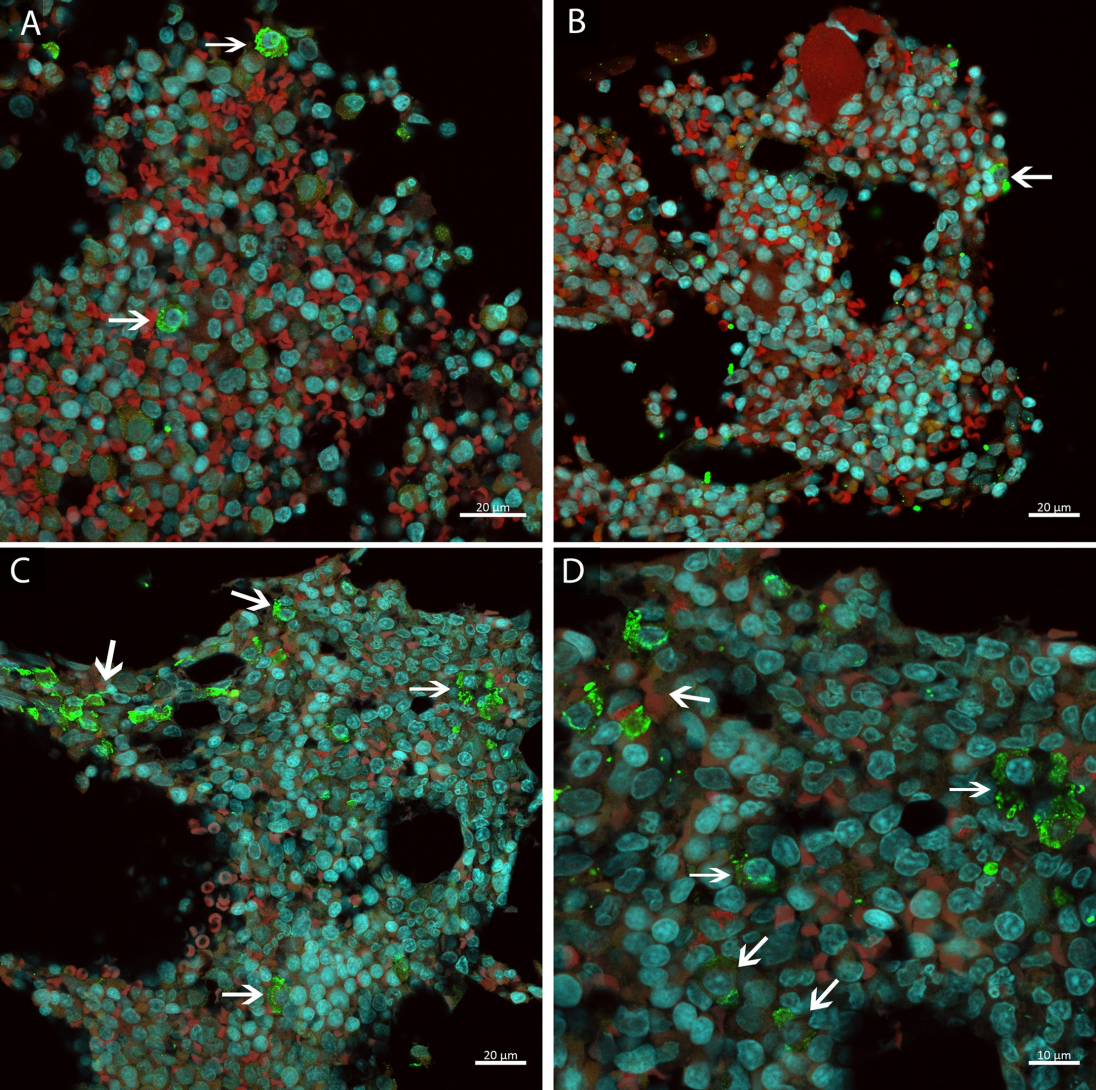
Frequency of HEV Ag-labelled bone marrow cells of cynomolgus monkeys experimentally infected with hepatitis E virus at 160 dpi. HEV antigen detection in: **(A-B)** monkeys with acute hepatitis E (1–2 cells / image field); **(C-D)** monkey with chronic hepatitis E (≥ 8 cells / image field). HEV antigen detection (→) in green, nuclei stained with DAPI in blue and erythroid cells and background in red.

HEV Ag was also detected by immunofluorescence in AC10 BM biopsy (Fig 2E). However, this animal did not show any apparent BM histological change (Table 4). Comparatively, frequency of HEV Ag-labelled cells was higher in the animal infected chronically (≥ 8 cells / image field) than in the animals infected acutely and naturally (1–2 cells / image field) (Fig 7A–7D).

## Discussion

Our study reports for the first time detection of HEV Ag in bone marrow (BM) cells from non-human primates (NHP). Experimentally infected animals, with chronic and acute hepatitis E, presented incipient histological signs, suggesting BM hyperactivation and dysfunction, characterized by vacuolization in endosteal cells, with some fields suggesting displasic focal disease. Medical research studies described severe aplastic anemia in association with parvovirus B19 (B19V), hepatitis A virus (HAV) and hepatitis C virus (HCV) infections [32–34]. Besides, recent studies described association between endosteal niche and loss of hematopoiesis homeostasis [35, 36].

According to our results, BM can be considered a site of viral persistence since detection of HEV Ag and dsRNA, a replicative marker, occurred at 160 dpi in acutely HEV infected monkeys, even though viral clearance in serum, feces, and liver had occurred within 56, 42, and 69 dpi, respectively.

HEV Ag was also detected in BM cells from the naturally infected animal, however, in absence of histological changes. Viral replication was confirmed, in both acute and chronic animals, by detection of dsRNA by immunostaining [37]. Unfortunately, HEV RNA detection by RT-PCR could not be performed in BM biopsies due to the limited amount of material available.

The monkey infected naturally, as well as the three NHP with acute hepatitis E, showed rare HEV Ag-labelled cells. On the other hand, in the chronically infected animal, a high number of those target cells was observed. A possible deactivation of immune system cells caused by immunosuppressive treatment with tacrolimus could explain such increased number of HEV Ag-positive cells persisting in BM [38, 39]. Adverse effects induced by tacrolimus long-term therapy associated to immune deactivation, such as: viral persistence and chronic hepatitis has been described [12]. The hemolytic-uremic syndrome may occur after administration of tacrolimus [40]. Moreover, microangiopathic hemolytic anemia and thrombocytopenia ─ rare, but potentially severe complications due to the use of immunosuppressive therapy ─ were not observed in our tacrolimus treated monkeys [41].

In order to characterize HEV target cells, it would be necessary to carry out phenotypic characterization. In our study, viral inclusions were not observed in hematoxylin and eosin staining. The use of immunostaining allowed the observation that detection of HEV Ag labeled cells were compatible with immature myeloid cells or even stem cells, both topographically and morphologically (large cells with a large nucleus and protruding nucleolus, with variable amounts of cytoplasm). Our findings corroborate with detection of HEV infection in post-alloHSCT in anti-HEV seronegative patients [42].

Detection of HEV Ag in BM cells also corroborates with evidence of transfusional HEV transmission and alerts to the possibility of transmission through BM transplantation. However, pathways of medullary infection are not yet confirmed [43, 44]. It is important to emphasize that in immunocompromised patients transfusional transmission of HEV, similar to other hepatotropic viruses, can worsen patient's clinical conditions [45, 46].

Despite low incidence of HEV infection as cause of acute hepatitis in post-alloHSCT, a high risk of developing chronic hepatitis with subsequent progression to liver failure, cirrhosis or even death has been associated with HEV infection in this risk group [23, 43]. Our results reinforce the need for systematic HEV screening of HSC donors with either a risk profile for HEV or abnormal liver tests, as suggested by other authors [20, 22, 23]. Besides, pre and post transplantation screening of patients should be considered once HEV target cells may present a late expression potential.

Besides, in July 2017, a naive colony-bred cynomolgus monkey (AC10) was found to be positive for HEV RNA. Serum samples obtained within a four years- monitoring period (November 2013; April 2015; April 2016; and July, August, and September 2017) were tested, with anti-HEV IgG seroconversion detected between April 2015 and April 2016. Although anti-HEV IgM was not tested, increasing levels of anti-HEV IgG in serial samples from April 2015 to July 2017 suggest recent infection (Fig 4). HEV RNA was detected in serum samples obtained in July and August 2017, concomitantly with decrease of anti-HEV IgG levels.

In general, the viremia period in cynomolgus monkeys is around 20 to 30 days [12, 47]. In our study, anti-HEV IgG seroconversion occurred between 2015 and 2016. Hence, detection of HEV RNA in serum samples from 2017 may suggest persistence of HEV infection, similar to that observed in a naturally infected Japanese monkey (*Macaca fuscata*) in a non-human primate colony in Japan, showing no evidences of immunosuppression [48]. Failure to detect HEV RNA in the sample retrospectively investigated in our study may be correlated with low viral load or long storage period, and successive freeze / thaw cycles. Another plausible assumption would be related to reactivation of HEV infection that has, so far, only described for alloHSCT recipients or antiviral-treated hepatitis C patients [11, 49]. Nevertheless, a viral reactivation was not observed in cynomolgus monkeys with acute hepatitis E, during the monitoring period of our NHP experiment, even after immunosuppression challenge with tacrolimus [12].

A high nucleotide identity was observed between HEV isolate obtained from the monkey (AC10) infected naturally and isolates from monkeys infected experimentally, as well as with inoculum obtained from a pig from a commercial farm located in Paraná state [12]. However, it is not possible to state the source of HEV transmission to the colony-bred animal as a full genome analysis would be required.

According to serological results, AC10 might have been infected at least three months after the end of the experimental study (February 2015). Thus, it is possible that contamination occurred due to protocol failures in waste management in the experimental area since the principal mode of HEV transmission is via the fecal-oral route. Moreover, as reported in a NHP colony in Japan, handlers can act as source of HEV transmission [48]. Unfortunately, serum samples of employees were not available in our study.

To the best of our knowledge, this is the first report of natural HEV infection among monkeys breed in South America. Together with the study conducted in the Japanese colony [48], our study highlights the need of continuing improvements and monitoring of preventive measures in non-human primate’s captive facilities.

Importantly, our results reinforce the hypothesis that HEV can be retained in BM cells even in resolving HEV infections with sustained viral response achieved spontaneously. Long-lasting NHP studies are necessary to follow up BM changes after the recovery phase of hepatitis E and its late hematological effects.

## References

[1] WHO. World Health Organization | Hepatitis E. 2016.

[2] Takahashi M, Nishizawa T, Nagashima S, Jirintai S, Kawakami M, Sonoda Y, et al Molecular characterization of a novel hepatitis E virus (HEV) strain obtained from a wild boar in Japan that is highly divergent from the previously recognized HEV strains. Virus Res. 2014;180:59–69. 10.1016/j.virusres.2013.12.014 24370869

[3] Woo PC, Lau SK, Teng JL, Tsang AK, Joseph M, Wong EY, et al New hepatitis E virus genotype in camels, the Middle East. Emerg Infect Dis. 2014;20(6):1044–8. 10.3201/eid2006.140140 24856611 PMC4036782

[4] Woo PC, Lau SK, Teng JL, Cao KY, Wernery U, Schountz T, et al New Hepatitis E Virus Genotype in Bactrian Camels, Xinjiang, China, 2013. Emerg Infect Dis. 2016;22(12):2219–21. 10.3201/eid2212.160979 27869607 PMC5189163

[5] Purdy MA, Harrison TJ, Jameel S, Meng XJ, Okamoto H, Van der Poel WHM, et al ICTV Virus Taxonomy Profile: Hepeviridae. J Gen Virol. 2017;98(11):2645–6. 10.1099/jgv.0.000940 29022866 PMC5718254

[6] Teshale EH, Hu DJ, Holmberg SD. The two faces of hepatitis E virus. Clin Infect Dis. 2010;51(3):328–34. 10.1086/653943 20572761

[7] Meng XJ. Hepatitis E virus: animal reservoirs and zoonotic risk. Vet Microbiol. 2010;140(3–4):256–65. 10.1016/j.vetmic.2009.03.017 19361937 PMC2814965

[8] Dalton HR, Izopet J. Transmission and Epidemiology of Hepatitis E Virus Genotype 3 and 4 Infections. Cold Spring Harb Perspect Med. 2018. 10.1101/cshperspect.a032144 PMC6211381 29530946

[9] Griffin SP and Nelson JE. Impact of a Clinical Solid Organ Transplant Pharmacist on Tacrolimus Nephrotoxicity, Therapeutic Drug Monitoring, and Institutional Revenue Generation in Adult Kidney Transplant Recipients. Prog Transplant. 2016; 26(4):314–321 10.1177/1526924816667950 27628498

[10] Wang Y, Zhou X, Debing Y, Chen K, Van Der Laan LJ, Neyts J, et al Calcineurin inhibitors stimulate and mycophenolic acid inhibits replication of hepatitis E virus. Gastroenterology. 2014; 146(7):1775 10.1053/j.gastro.2014.02.036 24582714

[11] Kamar N, Garrouste C, Haagsma EB, Garrigue V, Pischke S, Chauvet C, et al Factors associated with chronic hepatitis in patients with hepatitis E virus infection who have received solid organ transplants. Gastroenterology. 2011;140(5):1481–9. 10.1053/j.gastro.2011.02.050 21354150

[12] Gardinali NR, Guimaraes JR, Melgaco JG, Kevorkian YB, Bottino FO, Vieira YR, et al Cynomolgus monkeys are successfully and persistently infected with hepatitis E virus genotype 3 (HEV-3) after long-term immunosuppressive therapy. PLoS One. 2017;12(3). 10.1371/journal.pone.0174070 PMC5362194 28328941

[13] le Coutre P, Meisel H, Hofmann J, Rocken C, Vuong GL, Neuburger S, et al Reactivation of hepatitis E infection in a patient with acute lymphoblastic leukaemia after allogeneic stem cell transplantation. Gut. 2009;58(5):699–702. 10.1136/gut.2008.165571 19359434

[14] Halac U, Béland K, Lapierre P, Patey N, Ward P, Brassard J, et al Cirrhosis due to chronic hepatitis E infection in a child post-bone marrow transplant. J Pediatr. 2012;160(5):871–4. 10.1016/j.jpeds.2012.01.028 22341950

[15] Versluis J, Pas S, Agteresch H, de Man R, Maaskant J, Schipper M, et al Hepatitis E virus: an underestimated opportunistic pathogen in recipients of allogeneic hematopoietic stem cell transplantation. Blood. 2013;122(6):1079–86. 10.1182/blood-2013-03-492363 23794068

[16] Zylberman M, Turdo K, Odzak A, Arcondo F, Altabert N, Munne S. [Hepatitis E virus-associated aplastic anemia. Report of a case]. Medicina (B Aires).75(3):175–7. 26117610

[17] Shah SA, Lal A, Idrees M, Hussain A, Jeet C, Malik FA, et al Hepatitis E virus-associated aplastic anaemia: the first case of its kind. J Clin Virol. 2012;54(1):96–7. 10.1016/j.jcv.2012.02.002 22441030

[18] Kamar N, Selves J, Mansuy J-M, Ouezzani L, Péron J-M, Guitard J, et al Hepatitis E Virus and Chronic Hepatitis in Organ-Transplant Recipients. N Engl J Med. 2008. 10.1056/NEJMoa0706992 18287603

[19] Woolson KL, Forbes A, Vine L, Beynon L, McElhinney L, Panayi V, et al Extra-hepatic manifestations of autochthonous hepatitis E infection. Aliment Pharmacol Ther. 2014;40(11–12):1282–91. 10.1111/apt.12986 25303615

[20] Koenecke C, Pischke S, Beutel G, Ritter U, Ganser A, Wedemeyer H, et al Hepatitis E virus infection in a hematopoietic stem cell donor. Bone Marrow Transplant. 49 England2014 p. 159–60. 10.1038/bmt.2013.148 24056741

[21] Frange P, Roque-Afonso A, Neven B, Moshous D, Touzot F, Cavazzana M, et al Hepatitis E virus in hematopoietic stem cell donors: Towards a systematic HEV screening of donors? J Infect. 2015;71(1):141–4. 10.1016/j.jinf.2015.02.008 25727994

[22] O'Donghaile D, O'Flaherty N, Field S. Early hepatitis E infection in an unrelated hematopoietic progenitor stem cell donor. Bone Marrow Transplant. 2017;52(10):1471–2. 10.1038/bmt.2017.163 28714948

[23] Jaber M, Béland K, Rousseau C, Cellot S, Halac U, Alvarez F, et al Hepatitis E virus seroprevalence before hematopoietic SCT: a pediatric experience. Bone Marrow Transplant. 2014;49(6):857–8. 10.1038/bmt.2014.27 24637901

[24] Pischke S, Hiller J, Lutgehetmann M, Polywka S, Rybczynski M, Ayuk F, et al Blood-borne Hepatitis E Virus Transmission: A Relevant Risk for Immunosuppressed Patients. Clin Infect Dis. 63 United States2016 p. 569–70. 10.1093/cid/ciw309 27178472

[25] Kinugasa F, Nagatomi I, Ishikawa H, Nakanishi T, Maeda M, Hirose J, et al Efficacy of oral treatment with tacrolimus in the renal transplant model in cynomolgus monkeys. J Pharmacol Sci. 2008;108(4):529–34. 19098392 10.1254/jphs.08142fp

[26] Matthews KA, Tonsho M, Madsen JC. New-Onset Diabetes Mellitus After Transplantation in a Cynomolgus Macaque (Macaca fasicularis). Comp Med. 2015;65(4):352–6. 26310466 PMC4549682

[27] Mayer P. Notiz über Hämatein und Hämalaun. Zeitschrift für wissenschaftliche Mikroskopie und für mikroskopische Technik. 1903;20(409).

[28] Huang FF, Haqshenas G, Guenette DK, Halbur PG, Schommer SK, Pierson FW, et al Detection by reverse transcription-PCR and genetic characterization of field isolates of swine hepatitis E virus from pigs in different geographic regions of the United States. J Clin Microbiol. 2002;40(4):1326–32. 10.1128/JCM.40.4.1326-1332.2002 11923352 PMC140370

[29] Jothikumar N, Cromeans TL, Robertson BH, Meng XJ, Hill VR. A broadly reactive one-step real-time RT-PCR assay for rapid and sensitive detection of hepatitis E virus. J Virol Methods. 2006;131(1):65–71. 10.1016/j.jviromet.2005.07.004 16125257

[30] Drummond AJ, Suchard MA, Xie D, Rambaut A. Bayesian phylogenetics with BEAUti and the BEAST 1.7. Mol Biol Evol. 2012;29(8):1969–73. 10.1093/molbev/mss075 22367748 PMC3408070

[31] de Carvalho LG, Marchevsky RS, dos Santos DR, de Oliveira JM, de Paula VS, Lopes LM, et al Infection by Brazilian and Dutch swine hepatitis E virus strains induces haematological changes in Macaca fascicularis. BMC Infect Dis. 2013;13:495 10.1186/1471-2334-13-495 24148233 PMC3870956

[32] Weiler-Normann C, Hartl J, Weidemann S, von Pein UM, Fiedler W, Schramm C, et al Acute hepatitis as a prequel to very severe aplastic anemia. Z Gastroenterol. 2018;56(1):51–4. 10.1055/s-0043-121737 29316578

[33] Safadi R, Or R, Ilan Y, Naparstek E, Nagler A, Klein A, et al Lack of known hepatitis virus in hepatitis-associated aplastic anemia and outcome after bone marrow transplantation. Bone Marrow Transplant. 2001;27(2):183–90. 10.1038/sj.bmt.1702749 11281388

[34] Rauff B, Idrees M, Shah SA, Butt S, Butt AM, Ali L, et al Hepatitis associated aplastic anemia: a review. Virol J. 2011;8:87 10.1186/1743-422X-8-87 21352606 PMC3052191

[35] Galan-Diez M, Kousteni S. The osteoblastic niche in hematopoiesis and hematological myeloid malignancies. Curr Mol Biol Rep. 2017;3(2):53–62. 10.1007/s40610-017-0055-9 29098141 PMC5662025

[36] Duarte D, Hawkins ED, Akinduro O, Ang H, De Filippo K, Kong IY, et al Inhibition of Endosteal Vascular Niche Remodeling Rescues Hematopoietic Stem Cell Loss in AML. Cell Stem Cell. 2018;22(1):64–77.e6. 10.1016/j.stem.2017.11.006 29276143 PMC5766835

[37] Kyung-No S, Zhiguo L and Howard L. L. Double-Stranded RNA Is Detected by Immunofluorescence Analysis in RNA and DNA Virus Infections, Including Those by Negative-Stranded RNA Viruses. Journal of Virology. 2015 (18):9383–9392 10.1128/JVI.01299-15 26136565 PMC4542381

[38] Kannegieter NM, Shuker N, Vafadari R, Weimar W, Hesselink DA, Baan CC. Conversion to Once-Daily Tacrolimus Results in Increased p38MAPK Phosphorylation in T Lymphocytes of Kidney Transplant Recipients. Ther Drug Monit. 2016;38(2):280–4. 10.1097/FTD.0000000000000264 26606072

[39] Kannegieter NM, Hesselink DA, Dieterich M, Kraaijeveld R, Rowshani AT, Leenen PJ, et al The Effect of Tacrolimus and Mycophenolic Acid on CD14+ Monocyte Activation and Function. PLoS One. 2017;12(1):e0170806 10.1371/journal.pone.0170806 28122021 PMC5266297

[40] Tatapudi VS, Lonze BE, Wu M and Montgomery RA. Early Conversion from Tacrolimus to Belatacept in a Highly Sensitized Renal Allograft Recipient with Calcineurin Inhibitor-Induced de novo Post-Transplant Hemolytic Uremic Syndrome. Nephrol Dial. 2018; 19,8(1):10–19. 10.1159/000486158 PMC5836164 29594146

[41] Danesi R and Del Tacca M. Hematologic toxicity of immunosuppressive treatment. Transplantations Proceeds. 2004; 36:703–704. 10.1016/j.transproceed.2004.03.016 15110637

[42] Versluis J, Pas S, Agteresch H, de Man R, Maaskant J, Schipper M, et al Hepatitis E virus: an underestimated opportunistic pathogen in recipients of allogeneic hematopoietic stem cell transplantation. 2013;122(6):1079–86. 10.1182/blood-2013-03-492363 23794068

[43] Mirazo S, Ramos N, Mainardi V, Gerona S, Arbiza J. Transmission, diagnosis, and management of hepatitis E: an update. Hepat Med. 2014;6:45–59. 10.2147/HMER.S63417 24966702 PMC4051621

[44] Pischke S, Hartl J, Pas SD, Lohse AW, Jacobs BC, van der Eijk AA. Hepatitis E virus infection beyond the liver? J Hepatol. 2016. 10.1016/j.jhep.2016.11.016 27913223

[45] Kawatani T, Suou T, Tajima F, Ishiga K, Omura H, Endo A, et al Incidence of hepatitis virus infection and severe liver dysfunction in patients receiving chemotherapy for hematologic malignancies. Eur J Haematol. 2001;67(1):45–50. 11553266 10.1034/j.1600-0609.2001.067001045.x

[46] da Silva SG, Leon LA, Alves G, Brito SM, Sandes Vde S, Lima MM, et al A Rare Case of Transfusion Transmission of Hepatitis A Virus to Two Patients with Haematological Disease. Transfus Med Hemother. 2016;43(2):137–41. 10.1159/000441910 27226795 PMC4872048

[47] Aggarwal R, Kamili S, Spelbring J, Krawczynski K. Experimental studies on subclinical hepatitis E virus infection in cynomolgus macaques. J Infect Dis. 2001;184(11):1380–5. 10.1086/324376 11709779

[48] Yamamoto H, Suzuki J, Matsuda A, Ishida T, Ami Y, Suzaki Y, et al Hepatitis E virus outbreak in monkey facility, Japan. Emerg Infect Dis. 2012;18(12):2032–4. 10.3201/eid1812.120884 23171579 PMC3557886

[49] Rivero-Juarez A, Frias M, Lopez-Lopez P, de Los Angeles Risalde M, Brieva T, Machuca I, et al Hepatitis E Virus (HEV) Infection in Anti-HEV Immunoglobulin G-Carrying Patients After Successful Hepatitis C Virus Treatment: Reactivation or Reinfection? Clin Infect Dis. 2017;64(7):964–6. 10.1093/cid/cix004 28077520

